# Older Women and Successful Aging: Photovoice Reflections on Adaptation, Coping, and Connection

**DOI:** 10.1093/geroni/igaf122.2476

**Published:** 2025-12-31

**Authors:** Melinda Heinz

**Affiliations:** University of Northern Iowa, Cedar Falls, Iowa, United States

## Abstract

Seven participants in a Lifelong Learning Institute engaged in a photovoice project on successful aging. Participants aged 68-80, identified as White, and had professional careers that they were retired from. They met over three class sessions to 1) receive information and an overview of the study and 2) share photographs and narratives with each other and 3) learn more about current research in the field of successful aging after data collection had concluded. During the second session, participant discussions were recorded and all photographs and narratives were retained for this study. Braun and Clarke’s six-step thematic analysis approach was used for analysis. Steps include data familiarization, coding, early theme generation, refining themes, paring down themes, and results write up. Theme 1: Adapting and Coping revealed the importance of adapting to life challenges, with adjusting to widowhood as the most discussed life event. A subtheme on Challenging Yourself was noted and represented how participants enjoyed participating in lifelong learning and volunteering. Theme 2: Connections, included the diverse social resources that women had access to, such as friendships and connections with their faith/spirituality, as well as pets. Surprisingly, there was little discussion about the impact children or grandchildren had on successful aging. This finding differs from previous research and should be further explored in other groups of Baby Boomer women. It suggests that women may be more likely to consider resources outside of the home, not connected to traditional gender roles to age successfully and provides an interesting new direction for research.

